# OnabotulinumtoxinA to Prevent Chronic Migraine with Comorbid Bruxism: Real-World Data from the GRASP Study Group

**DOI:** 10.3390/toxins17110547

**Published:** 2025-11-03

**Authors:** Andreas A. Argyriou, Emmanouil V. Dermitzakis, Maria Chondrogianni, Aikaterini Foska, Dimitrios Rikos, Georgia Xiromerisiou, Panagiotis Soldatos, Pantelis Litsardopoulos, Michail Vikelis

**Affiliations:** 1 Headache Outpatient Clinic, Department of Neurology, Agios Andreas State General Hospital of Patras, 26335 Patras, Greece; pantelis84@hotmail.com; 2 General Clinic Thessaloniki, 54645 Thessaloniki, Greece; manolis.dermitzakis@gmail.com; 3 Second Department of Neurology, National and Kapodistrian University of Athens, School of Medicine, ‘Attikon’ University Hospital, 12462 Athens, Greece; mariachondrogianni@hotmail.gr (M.C.); dkfoska@gmail.com (A.F.); 4 404 Military Hospital, 41222 Larisa, Greece; rikosd@hotmail.com; 5 Neurology Institute, Mindful Mind, 55133 Thessaloniki, Greece; georgiaxiromerisiou@gmail.com; 6 Independent Researcher, 24100 Kalamata, Greece; soldatosp@gmail.com; 7 Glyfada Headache Clinic, 16675 Athens, Greece; mvikelis@headaches.gr

**Keywords:** chronic migraine, bruxism, comorbidities, OnabotulinumtoxinA, BoNTA

## Abstract

Background: This study, designed by the Greek Research Alliance for the Study of Headache and Pain (GRASP), sought to prospectively examine whether the treatment with two consecutive OnabotulinumtoxinA (BoNTA) cycles might improve the frequency and severity of chronic migraine (CM) with comorbid bruxism. We also explored whether the potential BoNTA-related alleviation of bruxism can directly influence the improvements in migraine efficacy outcomes. Methods: A total of 58 CM patients with comorbid bruxism at baseline, attaining two consecutive (quarterly given) BoNTA cycles, were studied. The changes in bruxism-related pain were assessed with the 0–10 numeric scale PI-NRS. Bruxism was clinically diagnosed using the self-report Bruxscreen-Q questionnaire. Any phenotypic changes in bruxism, according to Bruxscreen-Q, from baseline (T0) to the last efficacy evaluation follow-up (T1), were analyzed and then compared. Migraine-related efficacy and disability outcomes, mostly mean headache days (MHD), were also compared between T0 and T1. Results: BoNTA exerted significant improvements in bruxism-related pain, with PI-NRS median scores being significantly reduced from 7 at T0 to 3 at T1 (*p* < 0.001). The rates of masseter hypertrophy at T1 significantly dropped, compared to T0 (chi-square: 16; *p* < 0.001). Patients also self-reported significant improvements in the Bruxscreen-Q items at T1, compared to T0. At T1, 41/58 (70.7%) patients responded to BoNTA. The significant decrease in MHD frequency at T1 was positively correlated with improvements in bruxism-related pain severity (Pearson’s correlation: 0.710; *p* < 0.001). Conclusions: BoNTA exerts dual beneficial effects towards both the reduction of migraine frequency and the alleviation of bruxism-related pain and disability. Both of these effects seem closely interrelated in our study.

## 1. Introduction

Migraine is a frequent and clinically significant neurological condition characterized by recurring headaches of moderate or severe pain often accompanied by various debilitating symptoms, such as nausea, photophobia, phonophobia, and cutaneous allodynia. These headache-accompanying symptoms often exert additional influence on the daily activities and on the patients’ quality of life. The triggers for migraine may vary widely, including environmental factors, dietary habits, hormonal changes, and stress [1]. Patients with migraine often are unable to accomplish duties at work, social tasks, and personal relationships during migraine episodes, thus underlining the need for awareness and understanding of this condition [2].

The 2018 criteria of the International Classification of Headache Disorders—III classify migraines based on the frequency of monthly headache days (MHD) to episodic migraine (EM; less than 15 MHD) and chronic migraine (CM). CM is defined as headaches occurring for more than 15 days per month, with 8 of these having typical migrainous features or being triptan-responsive, for more than 3 months [3]. CM is generally much more severe and disabling than EM, due to increased symptom frequency and intensity, as a result of central sensitization [4]. CM patients may often experience an enormous emotional burden that is aggravated by stigma surrounding migraine, which can exacerbate feelings of isolation and insufficiency, but also withdrawal from social commitments and family interactions [5].

Migraine, and in general CM more than EM, is often associated with a variety of comorbidities, including, among many others, anxiety, depression, fibromyalgia, thyroidopathies, and obesity. Migraine comorbidities may aggravate its frequency and intensity and complicate management strategies. It was evident in the chronic study of epidemiology and migraine results (CAMEO) that especially psychiatric comorbidities significantly increase the inability related to headache in individuals suffering from migraine [6]. In addition, the study of symptoms and treatment of migraine in America (MAST) highlights the association between comorbid conditions and increased intensity and frequency of headache pain [7].

Bruxism appears to be a common and significant comorbidity, mostly in CM patients, as it can considerably affect the patients’ quality of life and treatment outcomes if it remains undiagnosed and untreated. Bruxism, characterized by the involuntary grinding of teeth or the tightening of the jaw while awake or during sleep, is associated with various factors, including psychological stress, muscle tension, and musculoskeletal disorders, such as temporomandibular joint disorders (TMDs) [8]. CM is often linked to similar etiological factors, including muscle tension and stress, which can exacerbate bruxism [9]. The interaction between these conditions suggests a potential shared pathophysiological mechanism, involving in particular the trigeminovascular system, which plays a crucial role both in headache and orofacial pain [10].

Management strategies for patients with CM and bruxism should address both the symptoms and the underlying causal factors. Given the shared mechanisms, treatment options, such as biofeedback and physiotherapy, have been suggested to alleviate the symptoms of the two conditions [11]. In addition, the use of oral devices, such as bruxism mouth guards worn during night sleep and/or during daily activities, can help reduce the impact of bruxism and, ultimately, decrease the frequency and intensity of headaches [12].

After its approval in 2010, OnabotulinumtoxinA (BoNTA) was added to the list of recognized treatments for adult CM prevention [13,14], while a recent systematic review pointed out that BoNTA treatment can substantially relieve migraine symptoms in many patients, offering a promising alternative to traditional, orally given, pharmacological therapies [15].

BoNTA prevents CM by inhibition of the release of neurotransmitters, in particular by blocking Snap-25 to reduce the release of peptides related to the calcitonin gene (CGRP), glutamate, and substance P from sensory nerve terminals [16]. Inhibition of Snap-25, which is a protein involved in synaptic transmission by forming part of the SNARE complex that mediates neurotransmitter release, could directly inhibit peripheral sensitization and neurogenic inflammation [17]. In addition, BoNTA can alleviate facial muscle tension, further contributing to its analgesic effects in preventing CM [18]. On clinical grounds, the experience from numerous real-world studies, including ours, clearly favors the long-term use of BoNTA over 3–5 years in CM prophylaxis [19,20,21,22].

Recently, the use of BoNTA has drawn attention as a possible therapeutic intervention for moderate/severe bruxism. A systematic review with meta-analysis found that BoNTA not only significantly reduced the frequency and severity of bruxism but also improved the patients’ quality of life [23]. Nonetheless, there is only scarce evidence in the literature [12] on whether BoNTA is able to exert dual beneficial effects in reducing the frequency of MHD, coupled with alleviation of the severity of bruxism in the setting of CM prophylaxis. Moreover, it remains not well known whether there is a direct relation between the improvement of bruxism and, in turn, the de-escalation from CM to EM with BoNTA treatment.

The aim of the current study, designed by the Greek Research Alliance for Studying headache and Pain (GRASP), was to examine whether the treatment with two (one every three months) consecutive BoNTA cycles might diminish the frequency and severity of CM with comorbid bruxism as well as explore whether the potential BoNTA-related alleviation of bruxism can directly influence the improvements in migraine efficacy outcomes.

## 2. Results

### 2.1. Characteristics of the Study Sample

We initially enrolled 58 consecutive BoNTA-naïve CM patients with comorbid bruxism at baseline. All (100%) of them received treatment with the second BoNTA course on a quarterly basis, as per protocol. The majority of patients included were female (*n* = 53; 91.4%), with a mean age of 46.5 ± 8.3 (range: 29–70) years. Migraine-related aura was seen in 11 patients (19%). Patients had failed a median of 5 (range: 3–8) prior preventatives, orally given, including beta or calcium channel blockers, antiepileptics, and antidepressants, while the median period since establishing the first migraine diagnosis was 26 years.

At baseline, significant comorbidity was noted. The majority regarded MOH and psychiatric comorbidities such as anxiety, depression, or mixed anxiety/depression, which were present in 55 (94.8%) and 30 patients (51.7%), respectively, while a minority presented fibromyalgia (*n* = 5; 8.6%) and TMDs (*n* = 5; 8.6%).

As predefined, either awake (3; 5.2%) or nocturnal bruxism (55; 94.8%) was present in all 58 patients, clinically phenotyped as either grinding or clenching. Masseter hypertrophy was evident in 46 (79.3%) patients. The subjects’ baseline clinical and epidemiological features are summarized in Table 1.

### 2.2. Effects of BoNTA on Bruxism Phenotype

**Figure 1:**
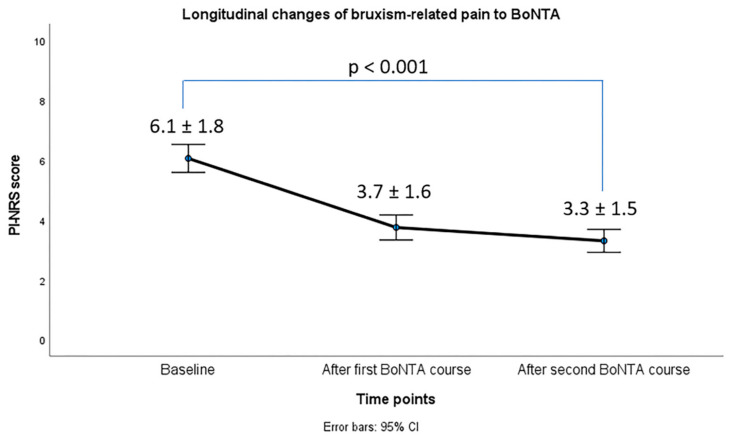
Longitudinal changes in bruxism-related pain, according to PI-NRS.

As can be seen in Figure 1, we observed significant improvements in bruxism-related pain. PI-NRS median scores were significantly reduced from 7 at T0 to 3 at T1 (*p* < 0.001).

The rates of masseter hypertrophy after injecting two consecutive BoNTA courses significantly dropped, compared to T0, as this was numerically disclosed in 46 (79.3%) patients at baseline and 25 (43.1%) patients at T1 (chi-square: 16; *p* < 0.001).

Patients also self-reported significant improvements in the Bruxscreen-Q items at T1 compared to T0. Indicatively, 32 (55.2%) patients (sum of often/always answers) reported nocturnal teeth clenching at T0 and 8 (13.8%) at T1. Awake teeth clenching was reported by 22 (38%) patients at T0 and 2 (3.4%) patients at T1. The same significant drop applied to the rates of nocturnal and awake teeth grinding between T0 and T1. Pain during awakening was reported at T0 from 17 (29.3%) patients and from 2 (3.4%) at T1.

### 2.3. Responder Rates and Effects of BoNTA on CM Efficacy and Disability Outcomes

At T1, the majority of treated patients (41/58; 70.7%) were categorized as treatment responders since their MHD had decreased by at least 50%. Of the 41 responders, 32 (55.2%) and 9 (15.5%) were able to maintain reductions in their MHD at T1 compared to T0 of >50% and >75%, respectively. These 41 BoNTA responders, in terms of migraine prevention, changed from MOH to non-MOH and de-escalated from CM to EM.

All effectiveness variables showed substantial improvements with BoNTA treatment, and a significant decrease (*p* < 0.001) was revealed at T1 when compared to baseline. Between T0 and T1, the mean MHD significantly decreased from 22.3 ± 4.3 to 12.1 ± 5.1. Monthly acute medication intake (MAI) was reduced from 18.6 ± 4.9 at T0 to 8.5 ± 5.8 at T1 (*p* < 0.001). The same applied for the median MHD with a peak headache intensity of ≥ 5. At T1 compared to T0, the median MIDAS and HIT-6 scores decreased by −43 and −9, respectively (*p* < 0.001), indicating a significant reduction in migraine-related impairment. Table 2 summarizes the changes in all disability and efficacy outcomes (median, range, z, *p*, and effect size values) between T0 and T1.

The decrease in MHD frequency at T1 was positively correlated with improvements in bruxism-related pain severity as measured by PI-NRS (Pearson’s correlation: 0.710; *p* < 0.001). The same was not applied for the percentage of >50% responders to BoNTA and lower masseter hypertrophy rates at T1, compared to T0 (chi-square: 8.99; *p* = 0.003).

All 41 treatment responders remained satisfied with the dual effect of BoNTA to both CM and bruxism alleviation and scored ≥5 on PGIC; specifically, 3 scored 5, 19 scored 6, and 19 scored 7 on PGIC.

### 2.4. Safety Analysis

There were no systemic adverse events or new safety signals linked to BoNTA. The majority of the adverse effects reported were mild and temporary, such as neck pain (*n* = 7), erythema at the injection site (*n* = 5), mild forehead ptosis (*n* = 3), and eyebrow elevation (*n* = 2). Unlike others, we have not experienced any adverse events after injecting masseters with BoNTA, such as reported elsewhere in the form of xerostomia, speech problems, masticatory weakness, dysphagia, bruising, asymmetry of the lower third of the face, and smiling changes [24].

## 3. Discussion

Patients with CM often report increased pain levels when bruxism is present, with evidence showing that this comorbidity contributes to the frequency and intensity of migraine [9]. The association between bruxism and primary headaches indicates that they can strengthen each other, as indicated in systematic review papers, which highlight the prevalence and implications of this comorbidity [25]. In addition, the mechanical deformation exerted on oral and facial structures during bruxism can further sensitize the brain, thus aggravating the clinical phenotype of migraine [26]. Hence, the quality of life in patients with CM is significantly affected by comorbid bruxism. Those who simultaneously experience the two conditions report higher levels of pain, anxiety, and depression compared to individuals with migraine alone [10]. The presence of bruxism also contributes to other symptoms, such as sleep disorders, which are essential to consider when evaluating the overall load of these comorbid conditions. Studies have shown that the presence of TMDs, often accompanying bruxism, can lead to an exacerbated quality of life in migraine patients [27].

Although there is some evidence to modestly support the effectiveness of BoNTA in TMDs and bruxism [28], there are currently quite a few studies to explore the potential of BoNTA in patients presenting CM and comorbid bruxism, by injecting BoNTA into the fixed sites according to the PREEMPT paradigm [13,14] but also masseter muscles [29]. To potentially add further evidence to the existing body of literature on the topic, we designed the current prospective study, wherein a well-characterized cohort of CM patients was treated with the fixed-site, fixed-dose PREEMPT strategy, to target the peripheral nerve endings of the cervical and trigeminal sensory system [30]. Masseter muscles were also injected bilaterally to potentially alleviate the comorbid CM bruxism.

We clearly demonstrate that BoNTA not only effectively prevented CM in 70% of enrolled patients but also significantly alleviated the pain related to bruxism, coupled with other significant improvements in the overall clinical phenotype of bruxism. However, the striking result of our study is considered to be the finding suggesting that the alleviation of bruxism is significantly associated with subserves, and possibly augments the core preventative effect of BoNTA in CM.

Our results are not directly comparable with the results of a previous publication, which, by using the PREEMPT injection protocol, coupled with injecting the masseter muscles, reported decreased MHD in patients with CM and comorbid TMDs [29]. Major methodological differences exist between our study and the later one that applied a retrospective study design to report that of 30 female CM patients enrolled, of whom 17 had concomitant TMDs, there were significant improvements in the groups with and without TMDs regarding the mean change of MHD at week 24. This study concluded that the comorbidity of TMDs is not a risk factor for migraine chronification. These findings are not in line with our results and directly contradict what is currently perceived to suggest that migraine and TMDs are two extremely common, overlapping pain conditions that significantly pose disability and impact the patients’ quality of life [31,32].

However, although the pathophysiology of these two disorders differs, there are shared physiopathological mechanisms, involving in particular the trigeminovascular system. As such, excessive jaw muscle activity in bruxism may sensitize trigeminal pathways to lower the threshold for migraine attacks [10]. In addition, CM is linked to abnormal brainstem pain processing, while bruxism may involve dysregulation of motor control in the brainstem as well. This overlap suggests a central mechanism involving hyperexcitability [33]. Neurotransmitter imbalance, i.e., dopaminergic dysfunction in bruxism, serotonergic in migraine, may contribute to both conditions, possibly explaining why they sometimes co-occur [8]. Finally, this dual beneficial BoNTA-related effect on both CM and bruxism can be clinically explained by the reduction in muscle tenderness and tension-type headache (TTH) days, which quite often coexist in CM patients, as denoted in the formal definition of CM, i.e., headache with ≥ 15 monthly headache days for at least 3 months, but typical migraine characteristics should occur on ≥ 8 monthly days [3]. Indeed, the majority of our patients (60%) had comorbid psychiatric comorbidities or fibromyalgia, which are both important elements of TTH. As such, the impact of psychological issues, which are critically involved in TTH, seems to be of great significance. This is because they may be the intermediate link between the various musculoskeletal symptoms due to the emotional tension that is downloaded in the form of muscle tension [34]. Moreover, it is suggested that the role of muscle bracing (the equivalent of clenching, but without tooth contact) during wakefulness is probably critical to the onset of symptoms, as the basic physiopathological pathway is that emotional load transfers to muscle tension, likely leading to symptoms due to muscle fatigue [35,36,37]. Nonetheless, further understanding the interaction between these conditions and shared mechanisms is crucial for the development of effective therapeutic approaches to those suffering from migraine and comorbid bruxism [38].

Efficacy aside, safety is also of paramount importance when introducing BoNTA as a modality of treatment for these comorbid conditions. In our study, no safety signals were disclosed, and adverse events were mild and transient. Although adverse effects are rarely reported, they may include localized pain and transient weakness in facial muscles [39], thus highlighting the importance of the thorough evaluation of the patient before proceeding. Informed consent might also be requested, considering individual variability in response to BoNTA, while long-term effects remain inadequately studied. In any case, research is needed to support standardized evidence-based integrated protocols in BoNTA administration for the management of comorbid migraine and TMDs with bruxism [24]. In line with the latter view are the findings of a recently published systematic review concluding that improved study protocols are required to provide direction for the clinical use of BoNTA to treat various orofacial neuropathic pain disorders [40].

Limitations in our study include the observational design we used and the moderate sample size that was assessed following two consecutive BoNTA sessions that were administered quarterly. Furthermore, there were no patient outcome measures to evaluate the patients’ psychological status and emotional aspects of their quality of life in our trial, which may have allowed for a more complete evaluation of BoNTA effects against the comorbid disorders. In spite of these limitations and the brief research period of two rather than three consecutive BoNTA courses before robustly establishing efficacy [41], we were able to show that BoNTA safely improved the bruxism load to migraine, and that all key migraine effectiveness measures showed clinically significant changes.

Among the strengths of our study should be included the use of the validated patient-reported outcome (PRO) tool, i.e., Bruxscreen-Q, to diagnose and monitor bruxism. Developed through rigorous methodology, the Bruxscreen-Q aims to identify the main symptoms associated with bruxism, emphasizing the importance of early diagnosis in assessing the possible presence of bruxism and its related jaw symptoms to improve patients’ management [42]. The use of this questionnaire not only facilitates the identification of individuals at risk but also helps physicians to plan personalized management strategies that can mitigate the negative consequences of bruxism, such as dental wear and myofascial pain. In addition, Bruxscreen-Q provides researchers comparable data on the prevalence and characteristics of bruxism in various populations [43]. These collective data allow more robust analyses and can lead to the identification of new treatment strategies, promoting innovation in bruxism management. One might claim that the Standardized Tool for the Assessment of Bruxism (STAB) might be the optimal tool to provide a multidimensional evaluation of bruxism status [44], and the lack of STAB use in the current setting might be another aspect limiting its generalization. Indeed, STAB was developed to aid in the collection of data on numerous facets, variables, and circumstances that are presently understudied in the discipline of bruxism. It is separated into two axes; axis A comprises the clinical (examiner report), instrumental assessment (technology report), and self-reported data on bruxism state and possible outcomes (subject-based report). The self-reported data (subject-based report) on conditions and circumstances that can contribute to the etiology or co-occurring conditions of bruxism is included in axis B [44]. Nonetheless, the face validity and on-field comprehension of the instrument are presently being evaluated. Moreover, the Bruxscreen-Q, used herein, shares similar metrics.

## 4. Conclusions

To conclude, the relationship between CM and comorbid bruxism is an important concern in the continuum of migraine management. Their shared mechanisms complicate both the clinical presentation and the results of the treatment, which has a negative impact on the overall quality of life. BoNTA appeared herein as a suitable treatment option. Considering that these dual beneficial effects seem closely interrelated, BoNTA appears to be a safe and effective treatment option to improve migraine outcomes for CM patients by effectively managing both the comorbid disorders.

However, it should be noted that pharmacological strategies aside, a comprehensive strategy that takes into account physical, psychological, and nutritional factors is essential to improving treatment outcomes and promoting greater health and well-being for those with CM who also have concomitant bruxism. To properly manage these complicated and overlapping disorders, it is necessary to conduct ongoing research to investigate standardized treatment paradigms. Moreover, there is a need to improve knowledge in the bruxism field and to use appropriate educational and terminological strategies, as outlined in a recently published report of an international consensus gathering with the goals of offering a dictionary of current definitions and outlining a plan for the subsequent actions necessary to improve comprehension of bruxism [45].

## 5. Materials and Methods

### 5.1. Study Design and Patients’ Selection

We prospectively enrolled adult CM patients who remained unresponsive or were intolerant to three previous oral preventatives, according to the approved indication and the national reimbursement policies. Patients were scheduled to be treated with BoNTA and were longitudinally followed up for two treatment cycles administered quarterly. The following inclusion criteria were applied: (i) a definite diagnosis of CM with or without aura or MOH, according to ICHD-III criteria [3]; (ii) CM patients with definite concurrent awake or nocturnal bruxism; (iii) were BoNTA-naïve; (iv) kept headache diaries and other migraine-related questionnaires, as instructed; and (v) keen to be contacted for additional phone interviewing to gather any further required data.

Throughout the trial, certified BoNTA injectors used the standard 0.5-inch 27G needle to deliver BoNTA (Botox^®^ 100UI/fl, Abbvie, Athens, Greece)on a quarterly basis, while closely following the fixed-dose (155 UI), fixed-sites PREEMPT paradigm [13,14]. An additional 45–60 UI was totally administered to the masseter muscles, 22.5–30 UI per side, depending on the volume of the masseter’s bulk. Variation in BoNTA units to treat concurrent bruxism depended on individual anatomy and needed to capture most of each masseter bulk from the anterior border to the oral commissure. The masseters’ bulk was clinically measured via direct visualization and palpation. Before determining the clinical outcomes of the intervention, participants received two consecutive treatment cycles, provided quarterly. The trial lasted eight months, including two months following the start of the second BoNTA course.

### 5.2. Primary and Secondary Objectives

Our primary objective was to evaluate the subjective improvement in the frequency and severity of bruxism, from baseline (T0—3-month pre-BoNTA period) to the period at two months after the administration of the last (second) course of BoNTA (T1). The changes in bruxism-related painful symptoms were assessed using the PI-NRS score, which is an 11-point pain intensity numerical rating scale ranging from 0 = no pain to 10 = worst possible pain [46]. The clinical diagnosis of bruxism was established with the use of the self-report Bruxscreen-Q questionnaire [47]. The self-reported Bruxscreen-Q contains the first part with six items related to bruxism, assessing the self-perceived presence of clenching and grinding while awake and during sleep. The second part contains an additional three items related to jaw symptoms and temporomandibular disorders (TMDs), evaluating both the pain and dysfunction aspects. Each item is scored on a six-point Likert scale (never; sometimes; regularly; often; always; and don’t know). Higher Bruxscreen-Q scores are in keeping with worse severity in bruxism [42].

Co-primary endpoints included i) the estimation of BoNTA-related ≥ 50% response rate at T1, compared to T0; ii) the crude percentage of patients experiencing < or >50% reduction in their MHD; (iii) changes in frequency monthly headache days (MHD) for CM patients; and (iv) changes in monthly intake of any abortive treatment (MAI) and changes in MHD with peak moderate/severe headache intensity of ≥ 5 in a visual analog scale (VAS), ranging from 0 to 10. To assess the changes in migraine-related disability, patients also longitudinally completed the Migraine Disability Assessment (MIDAS) and Headache Impact Test—6 (HIT—6). A higher score in MIDAS and HIT-6 indicates greater migraine-related disability [48,49,50].

The effects of BoNTA on both CM prevention and bruxism alleviation were assessed by estimating the changes in all the above primary and co-primary endpoints from T0 to T1. At T1, patients were also asked to rate their overall satisfaction with BoNTA with the use of the 7-point (1 stands for “no change” and 7 for “considerable improvement”) self-report “Patient Global Impression of Change” (PGIC) questionnaire [51]. 

### 5.3. BoNTA Safety Evaluation

The evaluation of safety was included among our secondary objectives. Safety evaluation was performed by encouraging patients to report any adverse events or severe adverse events (SAEs), either spontaneously or in response to direct questioning through phone contacts with patients at days 1–21 following every BoNTA infusion. The treating physician rated each adverse event as “related,” “possibly related,” “unlikely related,” or “unrelated” based on its association with BoNTA. Treatment-related adverse events (AEs) were defined as those with “related” or “possibly related” study drug associations. “Mild,” “moderate,” and “severe enough to lead to study drug discontinuation” were the ratings given to the severity of adverse events.

### 5.4. Statistical Analysis

Depending on the nature of each analyzed variable, descriptive statistics were produced by SPSS for Windows (version 27.0; SPSS Inc., Chicago, IL, USA). Between T0 and T1, changes in the primary and secondary efficacy variables’ values (mean or median, range, z, *p*, and effect size values) were recorded using the Wilcoxon rank test for paired data. Associations were established by calculating the Pearson’s correlation coefficient for ordinal data and using the chi-square test for categorical data. All tests were two-sided unless otherwise indicated, and *p* < 0.05 was considered significant.

## Figures and Tables

**Table 1:** Baseline demographic and clinical characteristics of enrolled patients prior to BoNTA exposure.

Participants *n* = 58 Variable	N (%)
**Gender** Females Males	53 (91.4) 5 (8.6)
**Age ± SD (range)**	46.5 ± 8.3 (29–70)
**Previous lines of prophylactic medications** Median value (range)	5 (3–8)
**Years with migraine diagnosis** Median value (range) **Psychiatric comorbidities** No Anxiety disorder Depression Mixed anxiety and depression disorder **Fibromyalgia** Yes No **Medication overuse headache** Yes No **Aura** Yes No **Temporomandibular joint disorders** Yes No **Masseter hypertrophy** Yes No	26 (5–50) 28 (48.3) 14 (24.1) 10 (17.2) 6 (10.4) 5 (8.6) 53 (91.4) 55 (94.8) 3 (5.2) 11 (19.0) 47 (81.0) 5 (8.6) 53 (91.4) 46 (79.3) 12 (20.7)

**Table 2:** Responder rates, changes in efficacy, and disability outcomes between baseline (T0—trimester before initiation of therapy) and after 2 courses of BoNTA treatment (T1) in 58 CM patients.

Median (Minimum–Maximum)					
Variables	Baseline	After Treatment with 2 BoNTA Cycles	z	*p* Value	Effect Size
Monthly headache days	22 15–30	11 3–28	−5.450	<0.001	0.70
Monthly days with peak headache intensity of at least 5/10 in VAS	17 6–30	6 1–25	−7.351	<0.001	0.86
Monthly days with intake of acute headache medication	20 8–30	6 2–25	−7.241	<0.001	0.85
MIDAS score	77 36–168	34 10–168	−6.944	<0.001	0.83
HIT-6 score	72 63–78	63 42–76	−5.398	<0.001	0.65
Response to BoNTA (%)					
<50%		17 (29.3)			
>50%		32 (55.2)			
>75%		9 (15.5)			
100%		0 (0)			

## Data Availability

The data that support the findings of this study are available from the corresponding author upon reasonable request.
